# Supplementary material for: Evaluation of change in trabecular bone structure surrounding dental implants by fractal dimension analysis and comparison with radiomorphometric indicators: a retrospective study
Source: PeerJ. 2022 Mar 22;10:e13145. doi: 10.7717/peerj.13145 (PMC8953503; doi:10.7717/peerj.13145)
Supplement: Supplemental Information 2 [file peerj-10-13145-s002.docx]

**(reference data obtained from : *Changes in the fractal dimension on pre- and post- implant panoramic radiographs , Sansare et al, 2012)***

**t tests -** Means: Wilcoxon signed-rank test (one sample case)

**Options:** A.R.E. method

**Analysis:** A priori: Compute required sample size

**Input:** Tail(s) = Two

Parent distribution = Laplace

Effect size d = 0.7142857

α err prob = 0.05

Power (1-β err prob) = 0.95

**Output:** Noncentrality parameter δ = 3.7588643

Critical t = 2.0529354

Df = 26.6929601

Total sample size = 29

Actual power = 0.9515962
