# Supplementary material for: Evaluation of change in trabecular bone structure surrounding dental implants by fractal dimension analysis and comparison with radiomorphometric indicators: a retrospective study
Source: PeerJ. 2022 Mar 22;10:e13145. doi: 10.7717/peerj.13145 (PMC8953503; doi:10.7717/peerj.13145)
Supplement: Supplemental Information 3 [file peerj-10-13145-s003.docx]

*-- Tuesday, October 12, 2021 -- 21:34:19*

**t tests -** Means: Wilcoxon signed-rank test (matched pairs)

**Options:** A.R.E. method

**Analysis:** Post hoc: Compute achieved power

**Input:** Tail(s) = Two

Parent distribution = Laplace

Effect size dz = 1.0246631

α err prob = 0.008493115

Total sample size = 30

**Output:** Noncentrality parameter δ = 6.8736490

Critical t = 2.7555126

Df = 44.0000000

Power (1-β err prob) = 0.9999627
